# Synergic quantum generative machine learning

**DOI:** 10.1038/s41598-023-40137-1

**Published:** 2023-08-09

**Authors:** Karol Bartkiewicz, Patrycja Tulewicz, Jan Roik, Karel Lemr

**Affiliations:** 1 Institute of Spintronics and Quantum Information, Adam Mickiewicz University, 61-614 Poznan, Poland; 2 Joint Laboratory of Optics of Palacký University and Institute of Physics of Czech Academy of Sciences, 17, Listopadu 12, 771 46 Olomouc, Czech Republic; 3 Poznan Supercomputing and Networking Center, Institute of Bioorganic Chemistry of the Polish Academy of Sciences, 61-704 Poznan, Poland

**Keywords:** Mathematics and computing, Physics

## Abstract

We introduce a new approach towards generative quantum machine learning significantly reducing the number of hyperparameters and report on a proof-of-principle experiment demonstrating our approach. Our proposal depends on collaboration between the generators and discriminator, thus, we call it quantum synergic generative learning. We present numerical evidence that the synergic approach, in some cases, compares favorably to recently proposed quantum generative adversarial learning. In addition to the results obtained with quantum simulators, we also present experimental results obtained with an actual programmable quantum computer. We investigate how a quantum computer implementing generative learning algorithm could learn the concept of a maximally-entangled state. After completing the learning process, the network is able both to recognize and to generate an entangled state. Our approach can be treated as one possible preliminary step to understanding how the concept of quantum entanglement can be learned and demonstrated by a quantum computer.

## Introduction

Generative adversarial network (GAN) machine learning is an intensely studied topic in the field of machine learning and artificial intelligence research^1^. While quantum machine learning research is attracting increasingly more attention both from the industry and the scientific community^2–27^, the quantum counterparts of GANs have been proposed in several recent papers works^28–30^. For example, in the proposal put forward by Dallaire-Demers and Killoran in Ref.^29^, the authors put much attention to specific circuit ansatz and discuss methods of computing gradients in specific types of variational quantum circuits. It is worth noting that the problem of computing gradients for variational quantum circuits is rather complex and can be also achieved by the parameter-shift rule^31,32^. In its general form, the proposal of Ref.^29^ includes sources of entropy (i.e., bath).

The idea behind GANs is rather simple, and it can be described with three circuits. The first circuit is the generator of real data ${\mathscr {R}}$, which is in principle an irreversible transformation depending on a value of a random variable $z_R$. In the case of quantum information this transformation at each instance takes the standard input state $|0\rangle$ and outputs a labeled random state $\rho _\lambda .$ A good example of such a generator is a painter who is asked to draw a cat (the label $\lambda$ is the animal here). There is not a unique deterministic way of drawing a cat, nor we know how to construct a painter from basic elements. However, we can train a stochastic quantum machine ${\mathscr {G}}$ to perform as generator ${\mathscr {R}}$ the same task by observing the output of ${\mathscr {R}}$ and its labels. However, this is not enough because ${\mathscr {G}}$ trained in this way, in general, will not be able to create new original instances, which can be labeled as $\lambda .$ Hence, an additional circuit ${\mathscr {D}}$ needs to be considered. This circuit is trained to distinguish between the samples $\rho _\lambda$ and the random output of ${\mathscr {G}},$ and it is referred to as *discriminator.*

The operation of ${\mathscr {D}}$ is optimal, if it assigns value 0 to states generated by ${\mathscr {R}}$ and value 1 to states generated by ${\mathscr {G}}.$ At the same time, the operation of ${\mathscr {G}}$ is optimal, if the cross-entropy between its output and states $\rho _\lambda$ is minimal while the discriminator is most likely to assign value 0 to the output of ${\mathscr {G}}.$ Thus, a GAN problem is solved by adversarial training of ${\mathscr {D}}$ versus ${\mathscr {G}}.$ The parameters of both the generator and discriminator can be found by numerical optimization or quantum gradient evaluation^29^ by dividing the training into rounds of adversarial optimization of both generator and discriminator. The circuits can perform an arbitrary computation as long as they are complex enough, admitting an arbitrary unitary operation and measurements on a number of ancillary qubits. However, similarly to classical artificial neural networks, choosing the appropriate architectures for specific concerns is a complex problem which is solved by trial and error. In quantum computing, this is even more so, because the lack of practical error correction limits the complexity of quantum circuits.

The quantum counterpart of GAN (i.e., QGAN) learning similarly to its classical analogue also finds Nash equilibrium of two player game, where one of the players generates some output and the second player (discriminator, ${\mathscr {D}}$) tries to tell if the output is generated by the first player (generator ${\mathscr {G}}$) or provided by an external source (${\mathscr {R}}$). This could be expressed as a min-max problem, where the statistical distance between the outputs of ${\mathscr {G}}$ and ${\mathscr {R}}$ is minimized over the strategies of the generator, whereas the distance between the outputs of ${\mathscr {D}}$ for ${\mathscr {G}}$ and ${\mathscr {R}}$, respectively, is maximized over the possible strategies of discriminator at the same time. In practice, this type of optimization if performed in rounds, and it is difficult to make the learning process stable. In a generative problem we do not have access directly to ${\mathscr {R}}$, but we can collect random samples generated by this source. However, we can formally treat ${\mathscr {R}}$ as a general multiqubit operation, where a specific unknown operation is selected according to an unknown probability distribution.

The general approach towards QGAN employs gradient-descent methods, as in the ansatz presented in Ref.^29^. In this standard QGAN it is impossible to apply the same sample from ${\mathscr {R}}$ to train both the discriminator and the generator due to the no cloning principle. Here, solve this problem by connecting the generator ${\mathscr {G}}$ and discriminator ${\mathscr {D}}$ in a single circuit. The intuition of the variational ansatz we present is that, we use the fact that we need to reach a conditional equilibrium state (i.e., an event when the states produced by ${\mathscr {G}}$ and ${\mathscr {R}}$ collapse on each other, yet at the same time the discriminator works at its peak performance) from the beginning of the training process. We train such a system by increasing the probability of a circuit state collapsing to this equilibrium state. This feature in some cases can lead to exponentially small value of the initial overlap for large dimentions. However, this can be circumvented by considering a problem specific.

In this new kind of machine learning for quantum GANs, where a conceptually simpler problem is being solved during the training than in a typical approach to QGAN. While QGAN requires setting the hyperparmeters responsible for training the generator and the discriminator in tuns, our approach does not require this. To introduce this approach we exploit time-reversal property of unitary transformations and properties of relative entropy. In particular, the approach can be understood intuitively by assuming the reversibility of the discriminator ${\mathscr {D}},$ which Hilbert space is the combined support space of the input state and a single-qubit decision register. We refer to this approach as *synergic quantum generative network* (SQGEN). The reversibility condition could be relaxed at the expense of raising the lower bound on the proposed cost function. In the extreme classical case the information on the input state is lost irreversibly in the discriminator and we cannot interpret the operation of SQGEN as conditioned on collapsing states produced by ${\mathscr {G}}$ and ${\mathscr {R}}$ on one another. Then, the cost function would be linear (instead of quadratic) in terms of the overlap between these states. This would impair the SQGEN ability to learn reproducing assemblages of density matrices instead of the mean density matrix describing the average output of ${\mathscr {R}}.$ In such a case, we loose the synergy between training ${\mathscr {G}}$ and ${\mathscr {D}}.$

The resulting variational quantum circuit can be trained using gradient methods, by means of parameter shift rules^31,32^ to compute partial derivatives of the cost function with respect to the circuit parameters. In many cases, it would be also practical to apply the Nelder–Mead method or similar algorithms to search for the optimal circuit parameters^33^. In our experimental demonstration of SQGEN we applied the Nelder–Mead method for optimizing the circuit. For our numerical simulations of the noiseless training of larger networks, we employed the BFGS algorithm, which is a gradient method. The simplex algorithms are better suited to deal with noisy function evaluations, which makes them a method of chosen for real experiments. The gradient method tends to work faster.

## Theoretical framework

**Figure 1:**
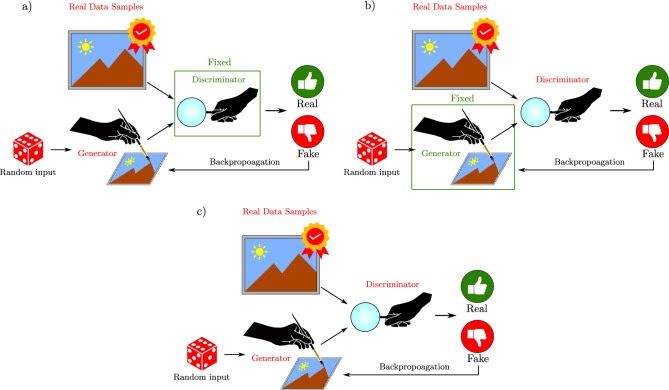
Panels (**a** and **b**) depict the training process of the generator and discriminator, respectively, in QGAN method. This approach involves iterative training alternating between (**a** and **b**) while maintaining a constant value of the generator or discriminator. On the other hand, in SQGEN method, as demonstrated in panel (**c**), the learning of the generator and discriminator involves cooperative interaction between them. Thus, the training process in SQGEN differs from that of QGAN).

As mentioned earlier, both SQGEN and QGAN comprise two main components: the generator ${\mathscr {G}}$ and the discriminator ${\mathscr {D}}$. The primary distinction between the two methods lies in their learning process. During training, the generator component aims to produce quantum states that cannot be differentiated from those in the training dataset, while the discriminator component seeks to accurately distinguish the generator’s quantum states from the training dataset. In QGAN, as depicted in Fig. 1a and b, the generator and discriminator’s learning processes alternate, with the discriminator’s value being fixed during the generator’s training and vice versa. In contrast, SQGEN’s learning process occurs concurrently, with the generator and discriminator components interacting simultaneously, as illustrated in Fig. 1c. Both approaches continue the iterative training process until the generator produces states that are indistinguishable from those in the training dataset.

**Figure 2:**
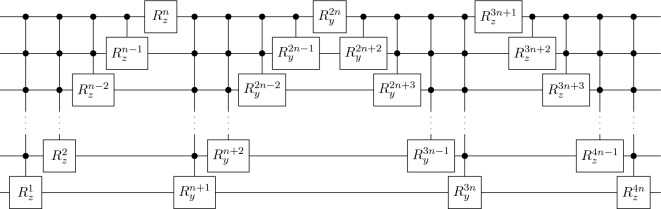
A decomposition of an arbitrary *n*-qubit unitary gate into *Z* and *Y* rotations (i.e., $R^n_z$ and $R^n_y$, respectively) controlled by multiple qubits as introduced in Ref.^34^. This circuit ansatz was used for software implementation of ${\mathscr {G}}$ and ${\mathscr {D}}$ gates for $n=1,2,3,4,5,6$. However, the final gates used in a real quantum device is obtained by automatically replacing the multiqubit controlled gates with a sequence of two- and single-qubit gates. In this ansatz, any *n*-qubit unitary gate is described by 4*n* parameters (i.e., rotation angles).

In our experiments and numerical simulations, we use the circuit ansatz of Möttönen et al. from Ref.^34^. This means that both ${\mathscr {G}}$ and ${\mathscr {D}}$ [i.e., $U_{z_D}$ from Eq. (6)] are implemented by a circuit block depicted in Fig. 2. We chose this particular ansatz because of its universality, uncomplicated implementation, and straightforward generalization to an arbitrary number of qubits. For a relatively small number of qubits, the exponential scaling in the number of CNOT gates does not constitute a problem. In higher dimensions, one can easily switch to a different ansatz, such as the so-called hardware efficient ansatz^35^ to avoid unfavorable scaling. In both cases, the number of parameters scales linearly with the number of qubits.

In “Methods” section, a detailed analysis of the discriminator’s precise principle and distinctions between the QGAN and SQGEN methodologies is presented. This section will delineate the process of determining the cost function that corresponds to each component utilized by the QGAN method, as well as the synergic anzatz that corresponds to SQGEN. Furthermore, we will introduce the quantum circuits essential in both approaches for determining the individual parameters.

### Quantum generative adversarial network

Here, we consider reversible (unitary) discriminators ${\mathscr {D}},$ which are provided with a generated state $\rho _{\lambda ,z_R}$ its label $\lambda ,$ a random variable $z_D,$ and a large enough ancillary Hilbert space to enable complex quantum computations. The $\lambda$ parameter serves as a label for generating data states^29^ and parameter $z_R$ is a random variable representing the unknown internal of its source, i.e., the state of generator ${\mathscr {R}}$. Note that $z_R$ is not accessible to the discriminator because the learning process must be independent of any knowledge on internal operation of the real generator ${\mathscr {R}}$. The task of the discriminator is to decide, for every input, if the input was indeed provided by generator ${\mathscr {R}}$ or not. The discriminator is trained only on a limited, but large, number of states $\rho _{\lambda ,z_R}$ and their labels. Note that in the classical ML the random variable is needed for the discriminator to make a decision if its input is real or fake, if the fakes are indistinguishable from the real inputs. In the quantum case, this is not necessary, as the collapse of a wave function of the discriminator output will achieve the same effect.

The third component is the circuit that is the model circuit of our generator ${\mathscr {G}}$ to be trained. This generator processes the same type of input as generator ${\mathscr {R}}$ and is provided with an independent random variable $z_G.$ We denote the output of this circuit $\sigma _{\lambda ,z_G} = |\psi _{\lambda ,z_G}\rangle \langle \psi _{\lambda ,z_G}|.$ The action of the generator is reversible as long as we know the value of the random variable $z_G.$ We assume that this is the case as this is a classical variable. We use random variables $z_G,z_R$ to represent the internal states of both the ${\mathscr {G}}$ and ${\mathscr {R}}$ generators, so we also get random states at the output of these gates. We train the generator ${\mathscr {G}}$ by observing the output of the source ${\mathscr {R}}$, but we cannot expect the output of ${\mathscr {G}}$ to be perfectly correlated with ${\mathscr {R}}.$ This is because, we only minimize the relative entropy of their outputs, defined as

1 $$\begin{aligned} S(\sigma _{\lambda ,z_G}||\rho _{\lambda ,z_R})= & {} \textrm{Tr}(\sigma ^2_{\lambda ,z_G}) -\textrm{Tr}(\sigma _{\lambda ,z_G}\log \rho _{\lambda ,z_R}) \end{aligned}$$

or in terms of Newton–Mercator series as

2 $$\begin{aligned} S(\sigma _{\lambda ,z_G}||\rho _{\lambda ,z_R})= & {} \langle 1-\rho _{\lambda ,z_R} \rangle + \langle ( 1-\rho _{\lambda ,z_R})^2 \rangle /2 + \langle ( 1-\rho _{\lambda ,z_R})^3 \rangle /3+ \ldots , \end{aligned}$$

where $\langle \rho _{\lambda ,z_R} \rangle = \langle \psi _{\lambda ,z_G}|\rho _{\lambda ,z_R} |\psi _{\lambda ,z_G}\rangle .$ By keeping only the first term of this expansion we are left with linear relative entropy $S_L$, which for random samples of $\sigma _{\lambda ,z_G}$ and $\rho _{\lambda ,z_R}$ becomes

3 $$\begin{aligned} S_L(\sigma _{\lambda ,z_G}||\rho _{\lambda ,z_R})= & {} 1 - \textrm{Tr}(\sigma _{\lambda ,z_G}\rho _{\lambda ,z_R}). \end{aligned}$$

Sample randomness (i.e., the statistics of $z_R$ and $z_G$), is required to place the linear entropy in the context of machine learning. The aim of a generative algorithm is, given samples $\rho _{\lambda ,z_R}$ prepare samples $\sigma _{\lambda ,z_G},$ which are statistically indistinguishable from new samples $\rho _{\lambda ,z_R},$ not used in the training. Thus, $S_L(\sigma _{\lambda ,z_G}||\rho _{\lambda ,z_R})$ should be minimized on average, i.e., over random samples denoted by $z_G$ and $z_R.$ To indicate such averaging, we drop the $z_G,z_R$ indices and from now we focus only on an average relative entropy. Note that relative entropy is in general jointly convex. In the linear approximation it is no longer the case, it is simply linear. This allows us to interpret $\rho _{\lambda }$ and $\sigma _{\lambda }$ as average density matrices of the states produced by the generators. For the generator ${\mathscr {G}}$ to mimic the source ${\mathscr {R}}$ correctly, it must also reproduce the probabilities of occurrence of the samples, not only to minimize the distance between the average states $\rho _{\lambda }$ and $\sigma _{\lambda }$. Therefore, using a discriminator is essential in our approach. While optimizing the generator ${\mathscr {G}}$, the discriminator ${\mathscr {D}}$ should reward a situation where a specific sample $\sigma _{\lambda ,z_G}$ is close a single sample of $\rho _{\lambda ,z_R},$ and penalize this otherwise. For this reason, the state of the discriminator must be independent of $z_G$ and $z_R.$ Moreover, assuming that a minimal achievable distance between $\rho _\lambda$ and $\sigma _\lambda$ has been reached, its cost function should be minimized if distributions of $z_G$ and $z_R$ are as similar as possible.

#### Generator ansatz

Linear entropy is directly measurable. Sometimes the second term in the expression is referred to as SWAP test. However, $S_L$ is alone is not enough to correctly train the generator. To demonstrate this, let us consider the following example, where random variables $z_G,z_R$ are given via probability distributions $p_\theta$ and $p_R$, respectively.

Thus, the mean linear entropy, or equivalently the cost function of the generator reads

4 $$\begin{aligned} J_G= & {} 1 - \sum _{z_G,z_R} p_\theta (z_G)p_R(z_R)\textrm{Tr}(\sigma _{\lambda ,z_G}\rho _{\lambda ,z_R}) =1-\textrm{Tr}(\sigma _{\lambda }\rho _{\lambda }), \end{aligned}$$

where $\sigma _{\lambda }=\sum _{z_G}p_\theta (z_G)\sigma _{\lambda ,z_G}$ and $\rho _{\lambda }=\sum _{z_R}p_R(z_R)\rho _{\lambda ,z_R}$ are mean outputs of the source and the generator.

The independence of $S_L$ on $p_\theta$ and $p_R$ can lead to the following case. Assume that we have at random two states, i.e., $\rho _0 = |0\rangle \langle 0|$ and $\rho _1 = |1\rangle \langle 1|$ with $p_\theta (0)=p_\theta (1)=1/2$. Now, we can reach the same value of relative entropy by using uniform sampling either from $\sigma _0=|+\rangle \langle +|$ and $\sigma _1 = |-\rangle \langle -|$ or from $\sigma _0=|0\rangle \langle 0|$ and $\sigma _1 = |1\rangle \langle 1|.$ This is as expected, as the two assemblages are indistinguishable merely by measuring overlap. For more details on quantum state discrimination, see the “Methods” section.

#### Discriminator ansatz

To resolve between the real and fake states, we need to go beyond a simple swap test and make use of a discriminator, which would calculate the probability of discriminating states $\rho _{\lambda ,z_R}$ and $\sigma _{\lambda ,z_G}.$ From the standard theory of optimal state discrimination we know that the probability of discriminating between two pure qubits can be expressed as $1-\cos ^2(\theta (\rho _{\lambda ,z_G})-\theta (\sigma _{\lambda ,z_G})).$ This can be easily understood in terms of the Mallus law, where qubits are encoded as single-photon polarization. In particular, one qubit is encoded as a linearly-polarized photon so that a polarizer can be set to transmit this photon. The second photon is transmitted with probability $\cos ^2(\theta (\rho _{\lambda ,z_G})-\theta (\sigma _{\lambda ,z_G})).$ Thus, the training of the discriminator corresponds to finding such a function $\theta$ that the value of $\cos ^2(\theta (\rho _{\lambda ,z_G})-\theta (\sigma _{\lambda ,z_G}))$ is minimized. This allows us to define the following cost function minimized by the discriminator and maximized by the generator, i.e.,

5 $$\begin{aligned} J_D= & {} 1-\sum _{z_G,z_R,z_D} p_\theta (z_G)p_R(z_R)g(z_D)\cos ^2(\theta _{z_D}(\sigma _{\lambda ,z_G})-\theta _{z_D}(\rho _{\lambda ,z_R})+\pi /2), \end{aligned}$$

where $g(z_D)$ is the probability of the discriminator having an internal state $z_D$. At the same time, we train the generator to produce an assemblage $\{\sigma _{\lambda ,z_G},p_R(z_G)\}$ which maximizes $\textrm{Tr}(\sigma _{\lambda ,z_G}\rho _{\lambda ,z_R})$ or $\cos ^2(\theta _{z_D}(\rho _{\lambda ,z_G})-\theta _{z_D}(\sigma _{\lambda ,z_G})).$

In order to associate this function with measurable quantities, we propose the following ansatz. We work on two registers containing the state to be processed by the discriminator, i.e., an ancillary qubit initialized as $|0\rangle$ and the processed state $|\psi \rangle$. The discriminator is now described by the following unitary operator performing a *y*-axis rotation on the ancillary qubit:

6 $$\begin{aligned} D= & {} \mathbbm {1} \otimes U_{z_D}|0\rangle \langle 0| U_{z_D}^\dagger +R_y(\theta )\otimes (\mathbbm {1} - U_{z_D}|0\rangle \langle 0| U_{z_D}^\dagger ), \end{aligned}$$

where $R_y(\theta )= \cos (\theta )\mathbbm {1} + i\sin (\theta )Y.$ Let $U_{z_D}|0\rangle =|\phi \rangle ,$ then $|\psi \rangle = \alpha |\phi \rangle + \sqrt{1-\alpha ^2}|\phi _\perp \rangle ,$ where $0\le \alpha \le 1$ and $\langle \phi |\phi _\perp \rangle =0.$ The probability of a state $|\psi \rangle$ being recognized as real by the discriminator is given as

7 $$\begin{aligned} p(\alpha )= & {} |\langle 0|\langle \psi | D | 0\rangle |\psi \rangle |^2 =|(1-\alpha ^2) \cos \theta + \alpha ^2|^2, \end{aligned}$$

where $p=1$ for $\alpha =1$ and arbitrary $\theta .$ In particular, the probability of a state $|\phi _\perp \rangle$ being recognized as real reads

8 $$\begin{aligned} p(0)= & {} |\langle 0|\langle \phi _\perp | D | 0\rangle |\phi _\perp \rangle |^2 = \cos ^2\theta , \end{aligned}$$

where $p=0$ for $\theta =\pi /2.$ Thus, we train the discriminator to have $\theta =\pi /2$ and $U_{z_D}$ which sets $|\phi \rangle \langle \phi |$ as close as possible to $\rho _{\lambda ,z_G}$ (i.e., $U_{z_D}|0\rangle \langle 0|U_{z_D}^\dagger \approx \rho _{\lambda ,z_G}$). From now on we will assume that $\theta =\pi /2$ unless stated otherwise.

It can be shown by direct calculations that the expression quantifying the difference between predictions of a discriminator for two different states reads

9 $$\begin{aligned} |p(\alpha )-p(\beta )|= & {} |\beta ^2-\alpha ^2|(\beta ^2+\alpha ^2)=|\sin (\theta _\alpha +\theta _\beta )\sin (\theta _\alpha -\theta _\beta )| \end{aligned}$$

where $\cos \theta _\alpha = \alpha ^2$ and $\cos \theta _\beta = \beta ^2.$ This difference is maximized if either $\beta =1$ or $\alpha =1$ i.e., the discriminator is set to maximize the *p* for a real state from assemblage $\{\rho _{\lambda ,z_R},p(z_R)\}.$ In this optimal case we arrive at the Mallus law for the discriminator, i.e.

10 $$\begin{aligned} |p(\alpha )-p(1)|= & {} \cos ^2(\theta _\alpha +\pi /2)=1-\alpha ^4, \end{aligned}$$

where $$\alpha ^2 =\cos \theta _\alpha = \langle \phi _{z_D}|\sigma _{\lambda ,z_G}|\phi _{z_D}\rangle$$.

The optimal settings for the discriminator are provided by minimizing in discriminability between assemblages $\{|\phi _{z_D}\rangle \langle \phi _{z_D}|,g(z_D)\}$ and $\{\rho _{\lambda ,z_R},p_\theta (z_R)\},$ i.e.,

11 $$\begin{aligned} J^*_D= & {} 1-\sum _{z_R,z_D} p_\theta (z_R)g(z_D)\cos ^2(\theta _\alpha ), \end{aligned}$$

where $$\alpha ^2 =\cos \theta _\alpha = \langle \phi _{z_D}|\rho _{\lambda ,z_R}|\phi _{z_D}\rangle$$ and $$|\phi _{z_D}\rangle = U_{z_D}|0\rangle$$.

If we reach the minimum of $J^*_D$ ($p=r$ and $\langle \phi _{z_R}|\rho _{\lambda ,z_R}|\phi _{z_R}\rangle =1$), then for the corresponding parameters of discriminator and assemblages $\{\rho _{\lambda ,z_R},p_\theta (z_R)\}$ consisting of orthogonal states, we can return to the original cost function

12 $$\begin{aligned} J_D= & {} 1-\sum _{z_G,z_R,z_D} p_\theta (z_G)p_R(z_R)g(z_D)\cos ^2(\theta _{z_D}(\sigma _{\lambda ,z_G})-\theta _{z_D}(\rho _{\lambda ,z_R})+\pi /2), \end{aligned}$$

where for a given assemblage $\{\sigma _{\lambda ,z_g},p_R(z_G)\}$ at minimum of $J^*_D$ we obtain $\theta _{z_D}(\sigma _{\lambda ,z_G})=\theta _\alpha$ and $\theta _{z_D}(\rho _{\lambda ,z_R})=(1-\delta _{z_D,z_R})\pi /2$. Here, $\alpha ^4 =\cos ^2\theta _\alpha = |\langle \phi _{z_D}|\sigma _{\lambda ,z_G}|\phi _{z_D}\rangle |^2=\textrm{Tr}(\sigma _{\lambda ,z_G}\rho _{\lambda ,z_D}).$ This function is now minimized over the parameters of the discriminator, regardless of the settings of the generator.

Such a discriminator is independent of the generator. However, if the input assemblage is unknown due to the no-cloning theorem, we cannot send the real states both to the generator and the discriminator operating in parallel. It is also impossible to train the generator and discriminator on the same set subsequently (as in traditional QGAN), as the states are destroyed during measurements. Thus, we need to design an alternative generative learning framework to QGAN.

#### Circuit for QGAN ansatz

**Figure 3:**
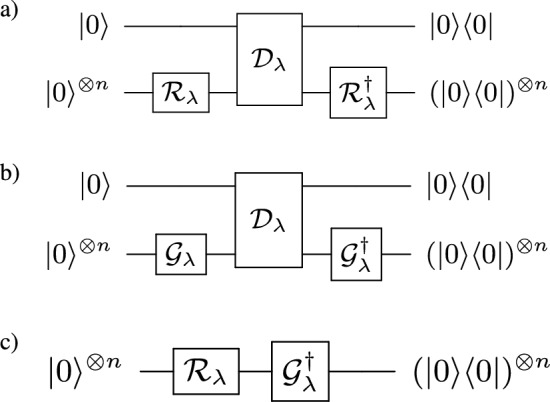
The circuits used for QGAN. (**a**) a circuit that evaluates the performance of ${\mathscr {D}}$ on ${\mathscr {R}}$ (computes *p*), (**b**) a circuit that evaluates the performance of ${\mathscr {D}}$ on ${\mathscr {G}}$ (computes *q*), and (**c**) a circuit that compares ${\mathscr {R}}$ with ${\mathscr {G}}$ (computes *F*). These circuits are used in rounds where for a given number of steps either ${\mathscr {D}}$ or ${\mathscr {G}}$ is optimized while keeping the parameters of the other fixed. A proper setting of this procedure requires a trial of error.

In the QGAN method, three crucial quantum circuits, shown in Fig. 3, are utilized for training the network. The first circuit Fig. 3a evaluates the performance of the discriminator ${\mathscr {D}}$ on real data generated by real data generator ${\mathscr {R}}$, and computes the probability of the ${\mathscr {D}}$ labeling the data as real, termed *p*. The second circuit Fig. 3b evaluates the performance of the discriminator on data, generated by the generator under training process ${\mathscr {G}}$, and computes the probability of the discriminator labeling the data as real, termed *q*. Finally, the third circuit Fig. 3c is responsible for comparing the distribution of real data and generated data and computing the fidelity *F*. These circuits are employed in alternating rounds where the parameters of either the discriminator or the generator are optimized while keeping the other fixed. The efficiency of this process relies heavily on proper settings, which often require trial and error experimentation to achieve optimal results. Overall, these three quantum circuits play a pivotal role in the QGAN method and provide the necessary framework for training a quantum generative adversarial network.

### Synergic quantum generative network

#### Synergic ansatz

As an alternative to the standard adversarial optimization, we propose minimizing a single cost function, i.e.,

13 $$\begin{aligned} J= & {} 1-\sum _{z_G,z_R,z_D} \left[ g(z_D)p_\theta (z_G)p_R(z_R)\cos ^2(\theta _{z_D})\right. \left. \textrm{Tr}(\sigma _{\lambda ,z_G}\rho _{\lambda ,z_R})\right] . \end{aligned}$$

If $\theta _{z_D}=0,$ this function reduces to $J=J_G$. If $\textrm{Tr}(\sigma _{\lambda ,z_G}\rho _{\lambda ,z_R})=1,$ the cost function *J* reduces to $J^*_D.$ The cost function can be interpreted as probability that the assemblages $\sigma$ and $\rho$ are distinguishable for a given setting of the discriminator. This quantity is minimized if both the generator and the discriminator are optimized simultaneously. If we optimize only the generator or the discriminator, there is always a place for improving *J* by optimizing the other. Finally, in order to improve the readability we plot an equivalent cost function

14 $$\begin{aligned} J= & {} 1-2\sum _{z_G,z_R,z_D} \left[ g(z_D)p_\theta (z_G)p_R(z_R)\cos ^2(\theta _{z_D})\right. \left. \textrm{Tr}(\sigma _{\lambda ,z_G}\rho _{\lambda ,z_R})\right] . \end{aligned}$$

Let us again assume that the source provides at random two states, i.e., $\rho _0 = |0\rangle \langle 0|$ and $\rho _1 = |1\rangle \langle 1|$ with $p_\theta (0)=p_\theta (1)=1/2$. Now, if we consider two configurations of the generator corresponding to equiprobable generation ($p_R(0)=p_R(1)=1/2$) of $\sigma _0=|+\rangle \langle +|$ and $\sigma _1 = |-\rangle \langle -|$ or $\sigma _0=|0\rangle \langle 0|$ and $\sigma _1 = |1\rangle \langle 1|,$ we can easily verify that for some configurations of the discriminator (corresponding to its optimal operation) the latter provides a lower value of *J*. This makes SQGEN to train the generator properly by introducing a discriminator, which is not the case when only considering generator.

#### Circuit for synergic ansatz

Let us for simplicity assume that all the probabilities *p*, *q*, *r* correspond to a single deterministic setting. The probabilities *q*, *r* are to be found by classical machine learning. The probability *p* is associated with the purity of the unknown assemblage $\{\rho _{\lambda ,z_R}),p(z_R)\}$. If for some $z_R$ we have $p(z_G)=1$ and $\rho _{\lambda ,z_R})$ is pure, then the assemblage is pure.

**Figure 4:**
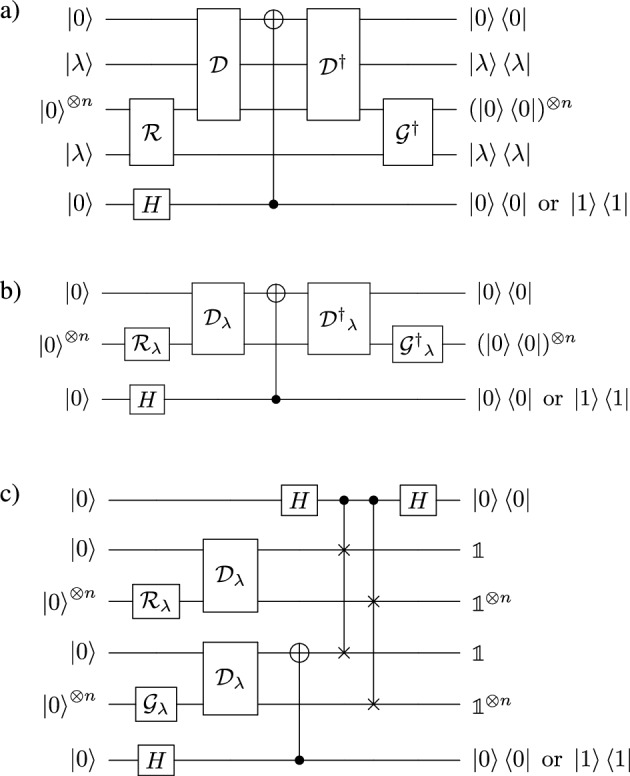
In the synergic quantum generative learning protocol, the probability of jointly postselecting the listed states is proportional to the value of the cost function (14). This means that the cost function reaches its maximum value if both the discriminator and the generator perform their tasks optimally. (**a**) State $|\lambda \rangle$ labels the class of the output of a generator. It is a control state that is not changed by the operation of source ${\mathscr {R}}$ or generator ${\mathscr {G}}.$ For a classical label $\lambda ,$ circuit (a) can be replaced with (**b**). In panel (**c**) we demonstrate an equivalent circuit inspired by a SWAP test^36^, where the measured quantity depends only on the rate of the projections of the first and the last qubits. Note that in the case of QGAN, in contrast to the synergic approach, one has to build (i) a circuit that compares ${\mathscr {R}}$ with ${\mathscr {G}}$, (ii) a circuit that evaluates the performance of ${\mathscr {D}}$ on ${\mathscr {R}},$ and (iii) a circuit that evaluates the performance of ${\mathscr {D}}$ on ${\mathscr {G}}.$ To compare SQGEN with QGAN we also include the bottom qubit in all the panels is measured in *Z* basis. Depending on the outcome, we include or ignore the existence of ${\mathscr {D}}.$ This allows to measure either the cost function or only source-generator fidelity. No hyperparamters are set by trial of error.

Now, instead of minimizing *J* we could equivalently maximize $1-J= \textrm{Tr}(\sigma _{\lambda ,z_G}\rho _{\lambda ,z_R})\cos ^2(\theta _\alpha ).$ Such a function can be measured directly in a single circuit. To this end, we propose connecting conjugated circuits to form a circuit that has ${\mathscr {D}}$ interfaced with its reverse of ${\mathscr {D}}$ with a conditional *X*-gate in between (i.e., Pauli $\sigma _x$ operation) in the first qubit as depicted in Fig. 4a. To reduce the complexity of this circuit, let us note that the labels marking the class to which a given state belongs to can be purely classical. This means that generator ${\mathscr {G}}$ and discriminator ${\mathscr {D}}$ can be controlled by a classical variable $\lambda$, which simplifies the quantum circuit from Fig. 4a to the one depicted in Fig. 4b. Note that the middle (generator) qubit in Fig. 4b can in general represent an arbitrary number of qubits, i.e., $\rho$ and $\sigma$ can be of arbitrary large Hilbert space.

The circuit in Fig. 4 with probability $\text{Tr}(\sigma _{\lambda ,z_G}\rho _{\lambda ,z_R})$ measures $|0\rangle$ for qubits other than the first ancillary qubit. This is equivalent to projecting the fake state $\sigma _{\lambda ,z_G}$ on the real state $\rho _{\lambda ,z_R}=|\psi \rangle \langle \psi |.$ Thus, by postselection, we measure the following value associated with cost function *J*, i.e.,

15 $$\begin{aligned} |\langle 0|\langle \psi | C_1 | 0\rangle |\psi \rangle |^2= & {} |-\sin (2\theta )(1-\alpha ^2) + \alpha ^2|^2=\cos ^2\theta _\alpha , \end{aligned}$$

where $\alpha ^2 =\cos \theta _\alpha = \langle \phi _{z_D}|\rho _{\lambda ,z_R}|\phi _{z_D}\rangle$ could be maximized equivalently for $\theta =\pi /2$ (discriminator regime) or $\theta =\pi /4$ (comparator regime), and $C_1=D^\dagger (X\otimes \mathbbm {1}) D,$ if the last qubit is projected on $|1\rangle$ or $C_0=\mathbbm {1}$ if the last qubit is projected on $|0\rangle .$ Then, we obtain $|\langle 0|\langle \psi | C_0 | 0\rangle |\psi \rangle |^2=1$ and we are left with a circuit independent of the discriminator parameters.

We have already discussed the discriminator regime $\theta =\pi /2.$ However, it is now apparent that we can also optimize the settings of the discriminator for $\theta =\pi /4.$ In such a case the probability of finding the first qubit in state $|0\rangle$ varies between $p(\alpha _{\textrm{max}})=1$ and $p(0)=1/2$. If for a given state the discriminator outputs $p=1,$ we know that the state was recognized as originating from the source. Thus, in the comparator regime it is convenient to use a value of $p'(\alpha )=2p(\alpha )-1$ and to interpret this value as a probability of recognizing the associated state as real, as in the discriminator regime. Now, we can observe that the measured probability $p(\alpha )$ compared against the probability *p*(0) of $|\phi _\perp \rangle$ being recognized as a real state becomes $p(\alpha )-p(0) = p'/2,$ hence the term comparator. This difference $p(\alpha )-p(0)$ is maximized while optimizing the discriminator. Thus, it is reasonable to introduce a cost function for a discriminator which could be easily interpreted in both regimes as the probability of a given state being properly associated with its origin (i.e., *G* or *R*), which reads $J_{D}=1-p'(\alpha )/2.$ In the discriminator regime $p'(\alpha )=p(\alpha )$ and in the comparator regime $p'(\alpha )=2p(\alpha )-1,$ where *p* is the measured quantity. Note that $J_{D}$ is optimized for the same parameters of discriminator in both regimes.

The complete circuit can be considered as working in two settings, depending on detecting $|0\rangle$ or $|1\rangle$ in the last qubit in Fig. 4b. In the latter case, the linear relative-entropy between the generator and the source can be measured by feeding states $\rho _{\lambda ,z_G}$ to the circuit and for the fixed values of $\lambda$ and $z_G,$ and consecutively measuring the rate at which the state of the generator line of the circuit is projected on $|0\rangle .$ However, this is only the case if the reversible discriminator returns $|1\rangle$ for a state generated by ${\mathscr {G}}$ and $|0\rangle$ for a state provided by ${\mathscr {R}}.$ The probability of this process is proportional to the rate at which the top line is projected onto $|0\rangle .$ Given that the top qubit is projected onto $|0\rangle ,$ the middle line measures the linear cross-entropy. In the opposite case (the decision qubit is detected to be in $|1\rangle$), the operation of the discriminator failed to be reversed and the detection rates of the middle line are meaningless. Hence, both the discriminator and the generator work at their best, if the joint detection rates of $|0\rangle$ in both top-most circuit qubits in Fig. 4b are maximized simultaneously. This is why we refer to the learning process as synergic learning. However, there exist solutions to this optimization problem, where the generator ${\mathscr {G}}$, taken separately from the discriminator, does not perform similarly to ${\mathscr {R}}.$ To address this issue, we consider the regime where only the similarity between ${\mathscr {G}}$ and ${\mathscr {R}}$ is maximized ($|0\rangle$ detected in the third qubit in Fig. 4b. More generally, we could consider the synergic learning as a process where both ${\mathscr {D}}$ and ${\mathscr {G}}$ are trained cooperatively, under the condition that ${\mathscr {G}}$ also is improving separately. To optimize the performance of the quantum setup, we propose to update its parameters using the Nelder–Mead algorithm or gradient descent to minimize the cost function (14).

To consider a possible ansatz for the discriminator, let us again consider the regime, where the *X* operation is active in the decision qubit. While maximizing the detection rates for $|0\rangle$ in the qubit generated state by varying the parameters of generator ${\mathscr {G}}$, we are making it less likely to detect $|0\rangle$ in the decision line. If the operations of ${\mathscr {G}}$ and ${\mathscr {R}}$ are identical, then gate *X* will flip the top qubit and could not achieve maximal two-fold detection rates of $|0\rangle$ in both qubits, unless we allow *D* to become a Hadamard gate *H*, conditioned on the similarity of ${\mathscr {R}}$ and ${\mathscr {G}}$ circuits. Note that, while maximizing the detection rates of $|0\rangle$ in the decision line by varying the parameters of the discriminator ${\mathscr {D}}$, in general, we do not necessarily decrease the value of relative entropy. If during the training the discriminator becomes a separable operation similar to $\sqrt{H}\otimes 1,$ and the generator ${\mathscr {G}}$ is very close to operating as ${\mathscr {R}}.$ Then, by optimizing ${\mathscr {G}}$ even further we would not influence the detection rate in the top qubit, i.e., the discriminator stops learning. In fact, the detection rate stops varying with ${\mathscr {G}}$ as soon as the operation ${\mathscr {D}}$ becomes separable. This suggests that inseparability of ${\mathscr {D}}$ is necessary to train the discriminator. Thus, it must be ensured during the design of ${\mathscr {D}}$ that its outcome in the decision qubit is strongly correlated with the generator qubits. This can be easily achieved by making the discriminator to consist of a *Y*-rotation controlled by the generator output qubits, targeting only the discriminator decision qubit. This rotation is set to $\pi /2$ to compute *p* and *q*,  and to $\pi /4$ in case of minimizing *J*. The discriminator should also admit arbitrary unitary transformations before the controlled operations. This guarantees that the output of a discriminator is state-dependent, and the optimization works as described above.

## Results

### Experimental single-qubit SQGEN

Let us consider a proof-of-principle experiment, where $\lambda$ labels the bases in which states are prepared. If $\lambda =x$ the generator ${\mathscr {R}}$ circuit ansatz, prepares at random state $(|0\rangle +|1\rangle )/\sqrt{2}$ or $(|0\rangle -|1\rangle )/\sqrt{2}.$ The eigenstates of the remaining Pauli matrices $\sigma _y$ and $\sigma _z$ are prepared if $\lambda =y,z.$ This in general requires feeding generators ${\mathscr {R}}$ and ${\mathscr {G}}$ with uncorrelated bivariate random variables $z_R$ and $z_G$ (baths), respectively. In addition, we require that the SQGEN performs equally well for all combinations of values of the random variables. Let us train a SQGEN with ${\mathscr {R}}$ set as a Hadamard matrix proceeded by $X^{z_R}$ operation (power of $z_R$), i.e., $\lambda =x$. To make the training process more transparent, let us focus on the special case of $z_G=0,$ only $(|0\rangle +|1\rangle )/\sqrt{2}$ is generated by ${\mathscr {G}}$.

**Figure 5:**
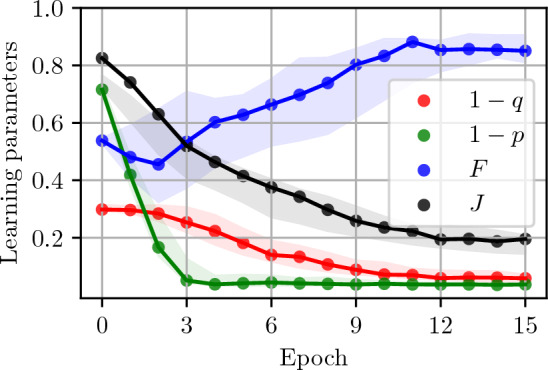
Experimental learning process performed with the quantum processor *ibmq*_*manila*^37^ using three qubits. The connected points represent the actual values measured in the training process performed as described in the main text. The shaded areas depict the range of values obtained in 100 Monte Carlo simulations with a fake provider *FakeManila()* delivered by Qiskit^38^ library. The simulation takes into account both shot-noise and transmon decoherence. This shaded area does not include all the points corresponding to the J cost function measurements performed on the real quantum processor. This means that the noise model provided for the *FakeManila()* by the manufacturer may be inadequate for circuits of the depth of order 27. This is not the case for the source-generator *F* and probability *p* (*q*) of the discriminator recognizing the source (generator) state as real.

In the experiment, we deal with finite numbers of shots, which can lead to random fluctuations in the measured values of the minimized cost function. To establish a sufficient number of shots, we analyzed the impact of this Poissonian noise on the experimental data. In the case considered, we used the Nelder–Mead algorithm because in the noise experiment, it gives better results than the gradient method, needing fewer steps to find the solution. From our numerical simulations, it follows that for our specific problem the training to perform well already for about 10^4^ shots for about 10^2^ evaluations of the cost function. When using more than 10^6^ shots the performance of Nelder–Mead algorithm further improves, reaching 70 cost function evaluations needed to find the minimum of the cost function. The speed of the convergence of this algorithm for this particular problem can be slightly improved by choosing a larger initial simplex. The requirements on the number of function evaluations and the number of coincidences make it feasible to implement conjugated SQGEN on a contemporary quantum computer. The results of the experiment are shown in Fig. 5.

We performed our experiments on *ibmq*_*manila* which is one of the *IBMQ*^37^ five-qubit alcon Processors (Falcon r5.11L) with Quantum Volume of 32. The parameters of the processor qubits, calibrated during the experiment, are shown in the Table 1.

**Table 1:** The parameters of the *ibmq*_*manila* processor’s qubits, calibrated at the time of the experiment.

Qubit	$T_1 (\upmu s)$	$T_2 (\upmu s)$	Frequency (GHz)	Anharmonicity (GHz)	Readout assignment error	CNOT error
0	97.769	108.828	4.9623	− 0.345	0.0265	0_1:0.005
1	178.823	76.082	4.838	− 0.345	0.026	1_2:0.010; 1_0: 0.005
2	130.887	24.069	5.037	− 0.343	0.034	2_3:0.007; 2_1:0.010
3	231.879	73.581	4.951	− 0.344	0.0194	3_4:0.006; 3_2:0.007
4	120.301	42.614	5.065	− 0.342	0.0204	4_3:0.006

Note that due to technical solutions used in IBMQ processors^37^ we cannot directly implement the circuit given in Fig. 4b. The processors, physically implement controlled-phase gates, controlled-not gates, and single-qubit rotations. This results in a circuit that performs 27 steps (circuit depth 27, 3 qubit circuit) before evaluating the cost function *J*. Independent 3 experiments were used to measure 16 values of real/fake state fidelity *F* (circuit depth 15, 1 qubit circuit), probability *p* of a real state (generated by ${\mathscr {R}}$) being classified by ${\mathscr {D}}$ as being real (circuit depth 11, 2 qubit circuit), probability *q* of a fake state (generated by ${\mathscr {G}}$) being classified as being real (circuit depth 11, 2 qubit circuit). These experiments were performed for parameter values found after each epoch of training.

For 20000 shots such circuit runs for 15 s per single cost function evaluation. For the random starting point used in Fig. 5, on average, we need 260 evaluations of the cost function to complete 15 training epochs (an epoch corresponds to 5 iterations of the Nelder–Mead algorithm). Our results show that the SQGEN training on a quantum processor (see Fig. 5) performs similarly as predicted by our numerical simulations. We did not use gradient-based approach here, as our experience shows that it is lest robust to experimental noise and because of this its convergence in many cases is worse than the Nelder–Mead methods.

The experimental results, shown in Fig. 5, demonstrate that SQGEN can can be implemented using the available quantum computers, even without applying error correction. However, to obtain our result we applied standard measurement and error mitigation, a method which corresponds to calibrating the detection part of the quantum computer.

To find the smallest number of shots needed for the learning process to complete, we have tested the proposed algorithm both on real quantum processor (*ibmq*_*manila*) and simulator(*simulator*_*statevector*) available to researchers via the IBMQ project^37^. Each evaluation of the circuit was performed on 8192 shots, which was found to be sufficient to limit the effect of Poisson noise. Due to the technical imperfections of these real devices, the algorithm converged only in about one half of the runs. It should be stressed out, however, that the user can always rerun the algorithm until it converges. One can observe that the algorithm converges to a non-zero value of the object function, which we also attribute to the experimental noise in the processor. Note that using the noiseless simulator, the algorithm converged on every attempt and the final object function was minimized below 0.001. This supports our finding that the algorithm is performing well, and the convergence difficulties are solely due to the noise in real presently available quantum processors.

### Comparison of QGAN and SQGEN: generating and recognizing a multiqubit entangled state

To illustrate the differences between the methods, let us consider generator ${\mathscr {R}},$ which prepares a maximally entangled (for $n>1$) *n*-qubit GHZ state $|\Psi \rangle =(|0\rangle ^{\otimes n}+|1\rangle ^{\otimes n})/\sqrt{2}.$ Thus, there is one possible value of $\lambda =e$. The goal of the QGAN and SQGEN training is to train generator ${\mathscr {G}}$ (i.e., find the optimal circuit parameters) without knowing the algorithm used by ${\mathscr {R}}$ nor its internal state $z_R$ by optimization of both the discriminator ${\mathscr {D}}$ and the generator ${\mathscr {G}}.$ The circuits used for QGAN and SQGEN are shown in Figs. 3a–c and 4b), respectively.

To compare the dynamics of the training process, we use (which will be discussed in more detail in “Methods” section) three figures of merit: (i) probability *q* of the fake (${\mathscr {G}}$-generated) state to be recognized as real by discriminator ${\mathscr {D}},$ (ii) probability *p* of the real state (${\mathscr {R}}$-generated) state to be recognized as real by discriminator ${\mathscr {D}},$ (iii) the distance between $D=1-F$ the ${\mathscr {G}}$-generated and ${\mathscr {R}}$-generated states (linear entropy). Here for noiseless numerical simulations we use a gradient descent method (i.e., BFGS), which guarantees at most as many function evaluations as the Nelder–Mead method. The learning process for both SQGEN and QGAN is performed for a fixed number of epochs. Each training epoch for SQGEN corresponds to a single iteration of BFGS algorithm used to minimize the cost function *J*. The relative number of iterations in QGAN is a hyperparameter that we tuned by trial of error. In the case of QGAN each epoch corresponds to one iteration of BFGS used to train the discriminator (to maximize a cost function proportional to $|p-q|$ and $p+q$) and a single iteration of BFGS to minimize *F*.

**Figure 6:**
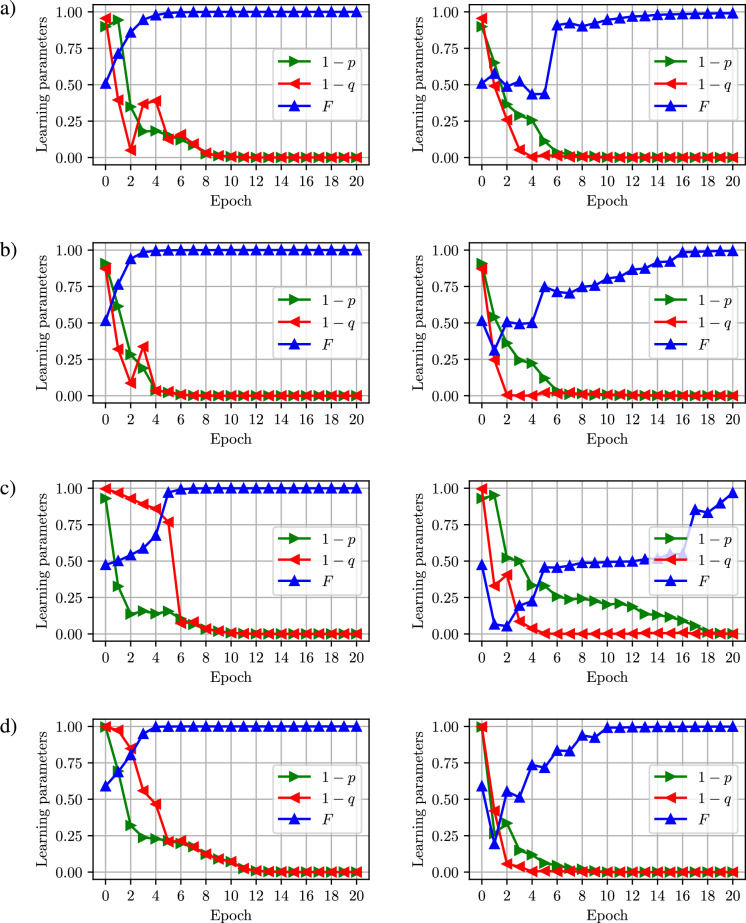
Comparison of the dynamics of the QGAN (left column) and SQGEN (right column) training process for the source providing *n*-qubit GHZ state. The sequence of panels corresponds to *n*,  i.e., (**a**) $n=2$, (**b**) $n=3$, (**c**) $n=4,$ (**d**) $n=5$. We do not plot *J* here, as QGAN has no counterpart of it. The plots illustrate the probability *p* of a source state being recognized as real by the discriminator, probability *q* of a generated state being recognized, and fidelity *F* comparing the trained generator and the source. These noiseless simulations were performed using *simulator*_*statevector*^37^.

In Fig. 6, showing the dynamics of the learning process optimized after a set of function calls corresponding to epochs, we see the comparison of QGAN and SQGEN results for the best achieved configuration of QGAN hyperparameters. More details on these simulations are summarized in Table 2.

**Table 2:** Comparison of performance of QGAN and SQGEN for 20 epochs of learning with BFGS optimizer for varied size of generated *n*-qubit GHZ state.

*n*	Feature	SQGEN	QGAN	
			Discriminator	Generator
1	Experiments per epoch	32.68	19.3	130.73
	Circuit depth	27	19	5
	Average time per epoch	0.93	1.52	
	Range of time per epoch	0.88–1.06	1.33–1.77	
2	Experiments per epoch	61.01	46.8	124.86
	Circuit depth	92	71	18
	Average time per epoch	10.87	9.97	
	Range of time per epoch	10.08–11.87	8.65–11.92	
3	Experiments per epoch	92	70.72	147.25
	Circuit depth	317	239	55
	Average time per epoch	52.87	39.80	
	Range of time per epoch	51.14–55.34	34.73–44.90	
4	Experiments per epoch	136.42	102	229.19
	Circuit depth	976	747	174
	Average time per epoch	229.44	174.34	
	Range of time per epoch	62.03–470.08	135.01–288.03	
5	Experiments per epoch	127.69	156.66	172.83
	Circuit depth	2404	1851	434
	Average time per epoch	516.45	515.85	
	Range of time per epoch	172.25–628.61	300.55–925.95	
6	Experiments per epoch	176.79	137.5	158.44
	Circuit depth	5385	4159	979
	Average time per epoch	1558.58	999.93	
	Range of time per epoch	443.82–2621.82	718.19–1148.19	

The total run time is given in seconds, and it may vary depending both on software and hardware. The run times here were obtained as averages over 5 runs (for various initial configurations) on a workstation equipped with Intel(R) Xeon(R) CPU X5690 @ 3.47GHz, using Python-based programs utilizing, e.g., qiskit, numpy, and scipy modules. The tabulated data corresponds to Fig. 6.

## Discussion

The proposed approach to generative quantum learning is conceptually different from the approaches described in Refs.^29,30^. Both approaches can solve an interesting problem, i.e., given samples of an entangled state, they can learn to generate the entangled states on their own. Moreover, the respective discriminators can be trained to detect the entangled state. However, from our numerical simulations it follows that for the same number of cost function calls, it is the SQGEN that will complete the training first.

For SQGEN, $n+1$ qubits are required to solve the problem ($n+2$ to also monitor *F* in addition to *J*), while for QGAN this value corresponds to as much as $3n+2$ qubits (this includes two sources ${\mathscr {R}}$). Even in the SWAP test based circuit, SQGEN requires fewer qubits than QGAN, i.e., $2n+4$ when monitoring *F*.

Our numerical investigations suggest that it takes fewer epochs for SQGEN to reach a stable probability values, and QGAN approach is slightly faster to settle on a high fidelity values. If the number of parameters is not too large and the hyperparameters of QGAN are set in an optimal way, the overall performance of SQGEN and QGAN is similar.

However, for GHZ states, QGAN appears to reach the optimal solution for a larger number of initial setup configurations and for a fixed number of training epochs. It is hard to state this with certainty due to a limited number of tested initial configurations. For each studied value of *n*, we found optimal generator and discriminator configurations using both approaches. For each $n>1$, we also found cases where either QGAN or SQGEN settled at suboptimal values. Note that the learning time depends on the initial input state, hence, the difference between the minimum and maximum values, shown in Table 2, is significant. As can be seen, this difference between the values occurs in both methods.

## Conclusions

We have proposed a new, efficient approach towards generative quantum machine learning. We have tested the proposed SQGEN algorithm experimentally on a small-scale programmable quantum processor. The experimental results shown in Fig. 5 confirm the feasibility of implementing SQGEN on a NISQ device. We have also performed feasibility study for larger experiments. However, we observed that experimental noise for $n>1$ prohibited reaching the convergence of the optimization procedure within the observed number of training epochs.

In addition to being conceptually different from a QGAN, SQGEN in all the cases investigated numerically required fewer cost function evaluations (experiments) per training epoch than QGAN. Note that SQGEN computes only *J* and *p*, *q*, *F* are computed additionally at the end of each epoch to facilitate our comparison with QGAN. Running a stable QGAN optimization is hard, as one has to carefully tune number of rounds and other parameters for training the discriminator and generator. Thus, the computational overhead of QGAN is in practice even greater. However, when properly tuned QGAN can demonstrate some advantage over SQGEN depending on a problem dimensionality and the initial choice of circuit parameters. Both methods sometimes settle at suboptimal solutions. The proposed SQGEN might be a good choice, if we do not want to deal with finding adequate values of many hyperparameters. Finally, SQGEN in contrast to QGAN does not require two copies of ${\mathscr {R}},$ which is important due to the no-cloning principle.

Note that in our numerical simulations, we have investigated how a quantum computer could learn the concept of a GHZ state. After the training, the network is able both to recognize and to generate this state. A next interesting step in would be to extend the notion of GHZ state to an arbitrary entangled state to investigate how the concept of entanglement could be learned and understood by a quantum computer. Solving this problem would require combining the presented concepts and methods with possibly more sophisticated classical machine learning to deal with providing labels for multidimensional, multiparty entanglement.

At this point, it is also important to stress that SQGEN cannot be directly reduced to a simple SWAP test, which corresponds to measuring only linear entropy. A SWAP test has an advantage when we are dealing with a source delivering a single pure state, but for general assemblages it not be sufficient to properly train the generator. However, the depth of the proposed SQGEN circuit can be potentially reduced (depending on the particular circuit ansatz) by applying the circuit depicted in Fig. 4c. The multi-qubit controlled-SWAP gate can be composed of *n* standard controlled-SWAP gates (i.e., Fredkin gates). This itself adds to the total circuit depth and at the same time increases exponentially the Hilbert space, which makes it difficult to simulate such circuits. However, using the SWAP test approach can reduce the time needed to evaluate *J* on a quantum computer with respect to the sequential circuit studied here, but by no more than a half. The SWAP test approach to SQGEN could be applied to mitigate to some extent dissipation in real quantum devices by reducing the impact of decoherence, which accumulates over time.

To some extent, we can compare the operation of the SQGEN circuit presented in this paper to that of an uncompressed autoencoder. Just as in the case of the autoencoder, the encoder it is trained together with the decoder, so in the circuit discussed here we train the generator and discriminator together. Another common aspect is the optimization of state fidelity between the input and the output. The key difference between the autoencoder and the problem at hand, is that due to the negation gate appearing, the right side of the circuit (regarded as a decoder) shown in Fig. 4, cannot be interpreted as the inverse of its left side (treated approximately as an encoder).

The aim of a generative algorithm is to generate samples that fit the properties of the real samples without knowing the ground truth (probability density function) about how the real samples are prepared. This is not the same as memorizing the real samples and generating them. We demonstrate that our approach is able to reproduce the real quantum samples and to distinguish between similar and dissimilar samples. In our analysis the ground truth about how the real samples were prepared was relatively simple. Thus, we were able to demonstrate that SQGEN works as intended. Demonstrating that SQGEN can handle more complex data patterns requires additional research as is beyond the scope of this paper.

Finally, it is interesting to consider some analogies between SQGEN and kernel-based machine learning. The initial part of the circuit can be viewed as the state preparation step, whereas the second part (including the *X* gate) can be interpreted as kernel evaluation circuit. Now, the SQGEN ansatz, as in the case of kernel-based methods, can be understood as a procedure consisting of measuring Gram matrix elements. However, the main difference is that contrary to standard kernel-bases approaches, we are not interested in evaluating Garm matrix elements for a specific fixed feature map and specific pairs of points in the feature space. In our case, the kernel is generated by both parameters of the source state (associated with the generator circuit) and the parameters of the discriminator. The circuits parameters are variables that we optimize and not fixed points in the feature space. The points are given by the generator and the source. Thus, in the variational circuit, we search for such a kernel that minimizes *J* with respect to circuit parameters. However, the circuit parameters appear with some weights which must be found by a classical algorithm, as in the case of standard applications of kernel methods.Thus, the SQGEN circuit could be considered as a generative kernel learning method which are being currently studied as a promising tool for generative learning^39^.

## Methods

### Quantum state discrimination

**Figure 7:**
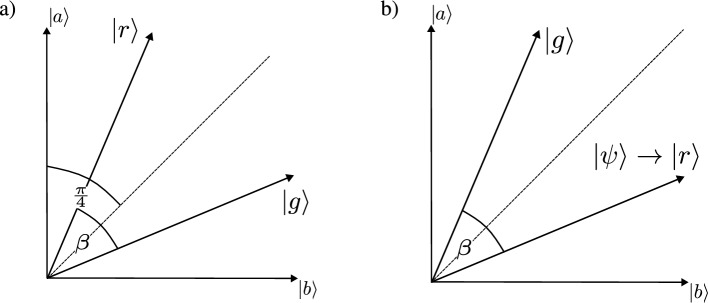
Geometric interpretation of state-discrimination strategies. Standard measurement-based approach in basis basis $|a\rangle ,|b\rangle$ (**a**) is compared to (**b**) discriminator-based approach used in SQGEN. The states to be discriminated are $|g\rangle$ and $|r\rangle .$ The internal state of the discriminator associated with the optimal discrimination is denoted as $|\psi \rangle$. The discriminator is trained to find an optimal section of a Hilbert space supporting $|g\rangle$ and $|r\rangle$, where the overlap $|\langle r|\psi \rangle |^2$ is to be maximal.

The main difference between QGAN and SQGEN approaches stems from the particular strategies applied for the state discrimination^40^ performed by the discriminator network, i.e., the interpretation and application of the performed measurements.

As an introduction to state discrimination, let us assume that we want to distinguish between two states $|g\rangle$ and $|r\rangle$ containing information on the output of a generative network and the real data, respectively. These states regardless of their dimension can be represented as unit vectors on a plane. The angle between these two vectors is given as $\beta .$ The standard approach to state discrimination is finding such basis $|a\rangle ,|b\rangle ,$ where the states to be discriminated are expressed as $|r\rangle =\cos (\pi /4-\beta /2)|a\rangle + \cos (\pi /4+\beta /2)|b\rangle$ and $|g\rangle =\cos (\pi /4+\beta /2)|a\rangle + \cos (\pi /4-\beta /2)|b\rangle .$

Then, the probability of these two states being discriminated via von Neumann measurements reads $p_{a,b}= |\langle g|a\rangle |^2 |\langle r|b\rangle |^2 + |\langle g |b\rangle |^2 |\langle r |a\rangle |^2.$ This expression can be reduced to $p_{a,b}=(1+\sin ^2\beta )/2.$ This is the case QGAN training, where the optimization of discriminator consists of increasing the probability of projecting states $|r\rangle$ and $|g\rangle$ onto a state $|\psi \rangle$ co-planar with $|a\rangle$ and $|b\rangle$ while maximizing the angle $\beta$ between the discriminated states (i.e. finding the basis $|a\rangle ,|b\rangle$), see Fig. 7a. Sate is $|\psi \rangle$ given by a current configuration of the discriminator.

Instead of discriminating multidimensional states directly, we can introduce a single-qubit discriminator register initialized as $|0\rangle$. Now, a discriminator performs a controlled $R_y(\theta )$ on this register, where $R_y(\theta )$ is controlled by a given input of the discriminator ($|r\rangle$ or $|g\rangle$), i.e.,

16 $$\begin{aligned} R_y(\theta )\otimes |\psi \rangle \langle \psi | + \mathbbm {1}\otimes (\mathbbm {1}-|\psi \rangle \langle \psi |), \end{aligned}$$

where $\phi$ and $|\psi \rangle$ are parameters of the discriminator. Next, the register qubit is measured in z-basis, which yields for input $|r\rangle$ two outcomes, i.e., $-1$ with probability $p^{(-)}_{r} =\sin ^2\theta |\langle r| \psi \rangle |^2$ and $+1$ with probability $p^{(+)}_{r} =1-\sin ^2\theta |\langle r| \psi \rangle |^2.$ The probability of optimal discrimination is given as $p^{(-)}_{r}p^{(+)}_{g} + p^{(-)}_g p^{(+)}_r=(1+\sin ^2\beta )/2,$ if $|\psi \rangle =|r\rangle$ or $|\psi \rangle =|g\rangle$ and $\sin \theta =1.$ This situation is depicted in Fig. 7b.

Both QGAN and SQGEN train the discriminator to reach its optimal performance. The advantage of SQGEN is that it automatically sets its internal pointer $|\psi \rangle$ state to $|r\rangle$, i.e., only the cases, where $|r\rangle$ collapses onto $|\psi \rangle$ and $|g\rangle$ collapses onto the support space of $\mathbbm {1} - |\psi \rangle \langle \psi |$ are counted as the relevant events. In case of QGAN the discriminator has to learn how to discriminate between $|r\rangle$ and $|g\rangle$ having access to only one of them at a time. This means that it performs superfluous computations that are needed for establishing a reference frame for the discrimination process. The details of the discriminator training for SQGEN together with the discriminator ansatz are discussed further in the text.

The discriminator works at its best when the probability of state discrimination is maximized. We can maximize this probability instead of the difference of rates of assigning Real/Fake label to a sample delivered by ${\mathscr {R}}$ or ${\mathscr {G}}$, as it is done in the standard GAN. This probability will be lowered, if the similarity between the samples given by ${\mathscr {R}}$ or ${\mathscr {G}}$ is increased, as it happens to the aforementioned difference of rates.

## Data Availability

The datasets used and/or analysed during the current study available from the corresponding author on reasonable request.
